# Association between the psoas muscle index and hospitalization for pneumonia in patients undergoing hemodialysis

**DOI:** 10.1186/s12882-021-02612-7

**Published:** 2021-11-27

**Authors:** Kosei Yamaguchi, Mineaki Kitamura, Takahiro Takazono, Shuntaro Sato, Kazuko Yamamoto, Satoko Notomi, Kenji Sawase, Takashi Harada, Satoshi Funakoshi, Hiroshi Mukae, Tomoya Nishino

**Affiliations:** 1 Department of Nephrology, Nagasaki University Graduate School of Biomedical Sciences, Nagasaki, Japan; 2 Nagasaki Renal Center, Nagasaki, Japan; 3 Department of Infectious Diseases, Nagasaki University Graduate School of Biomedical Sciences, Nagasaki, Japan; 4 Department of Respiratory Medicine, Nagasaki University Hospital, Nagasaki, Japan; 5 Clinical Research Center, Nagasaki University Hospital, Nagasaki, Japan; 6 Department of Respiratory Medicine, Nagasaki University Graduate School of Biomedical Sciences, Nagasaki, Japan

**Keywords:** Hemodialysis, Muscle mass loss, Pneumonia, Psoas muscle index

## Abstract

**Background:**

Although muscle mass loss and pneumonia are common and crucial issues in hemodialysis (HD) patients, few reports have focused on their association, which remains unclear. This study assessed the association between skeletal muscle mass and the incidence of pneumonia in HD patients using the psoas muscle index (PMI).

**Methods:**

This retrospective study included 330 patients on HD who were treated at a single center between July 2011 and June 2012. The observation period was between July 2011 and June 2021. Demographic, clinical, and HD data were collected, and the associations between PMI and hospitalization due to bacterial pneumonia were evaluated using Cox proportional hazards models adjusted for patients’ background data. Additionally, the correlation between patient characteristics and PMI was evaluated using multivariable linear regression.

**Results:**

Among 330 patients (mean age, 67.3 ± 13.3; 56.7% male; median dialysis vintage 58 months, (interquartile range [IQR] 23–124), 79 were hospitalized for pneumonia during the observation period (median observation period was 4.5 years [IQR 2.0–9.1]). The multivariable Cox proportional analysis, which was adjusted for age, sex, dialysis vintage, diabetes mellitus, and stroke history and considered death as a competing risk, indicated that decreased PMI/(standard deviation) was closely associated with the development of pneumonia (hazard ratio: 0.67, 95% confidence interval: 0.47–0.95, *p* = 0.03).

**Conclusions:**

Skeletal muscle mass was associated with the development of pneumonia in patients on HD and could be a useful marker for the risk of pneumonia.

**Supplementary Information:**

The online version contains supplementary material available at 10.1186/s12882-021-02612-7.

## Background

Infectious diseases are one of the main causes of death in patients undergoing hemodialysis (HD) [1, 2]. As the mean age of HD patients increases due to the aging global population [2], contemporary HD patients are tending to be more immune-deficient than those in the past. This is attributable to their clinical backgrounds, which may include leukocyte dysfunction, malnutrition, and frailty [3]. According to the United States Renal Data System registry and National Center for Health Statistics, pneumonia is the most common infectious cause of death in HD patients, with a mortality rate that is 14–16 times higher than that in the general population [4]. Further, a history of pneumonia in HD patients is associated with cardiovascular diseases and death [4]. Therefore, pneumonia, including aspiration pneumonia, is relevant to the management of HD patients and should be prevented to improve survival. However, reports on the risk of developing pneumonia in patients undergoing HD are scarce [5].

Muscle mass loss is common in HD patients [6, 7] and has adverse outcomes, including physical disability, poor quality of life, and death [8]. There are several known risk factors for infection, some of which have been reported to be associated with muscle mass. For example, dysphagia is the most common cause of pneumonia and is associated with skeletal muscle mass loss [9]. Further, malnutrition is associated not only with skeletal muscle mass loss [6] but also immune deficiency [10]. Conventionally, the nutritional status of HD patients is assessed according to the geriatric nutritional risk index (GNRI) [11] and the nutritional risk index (NRI) for HD patients [12].

Here, we hypothesized that skeletal muscle mass loss would be associated with the development of pneumonia. We investigated the association between skeletal muscle mass and pneumonia incidence in HD patients using the psoas muscle index (PMI), determined using abdominal computed tomography (CT) as an indicator of skeletal muscle mass [13]; its associated factors, including nutritional parameters, were also investigated.

## Methods

**Fig. 1:**
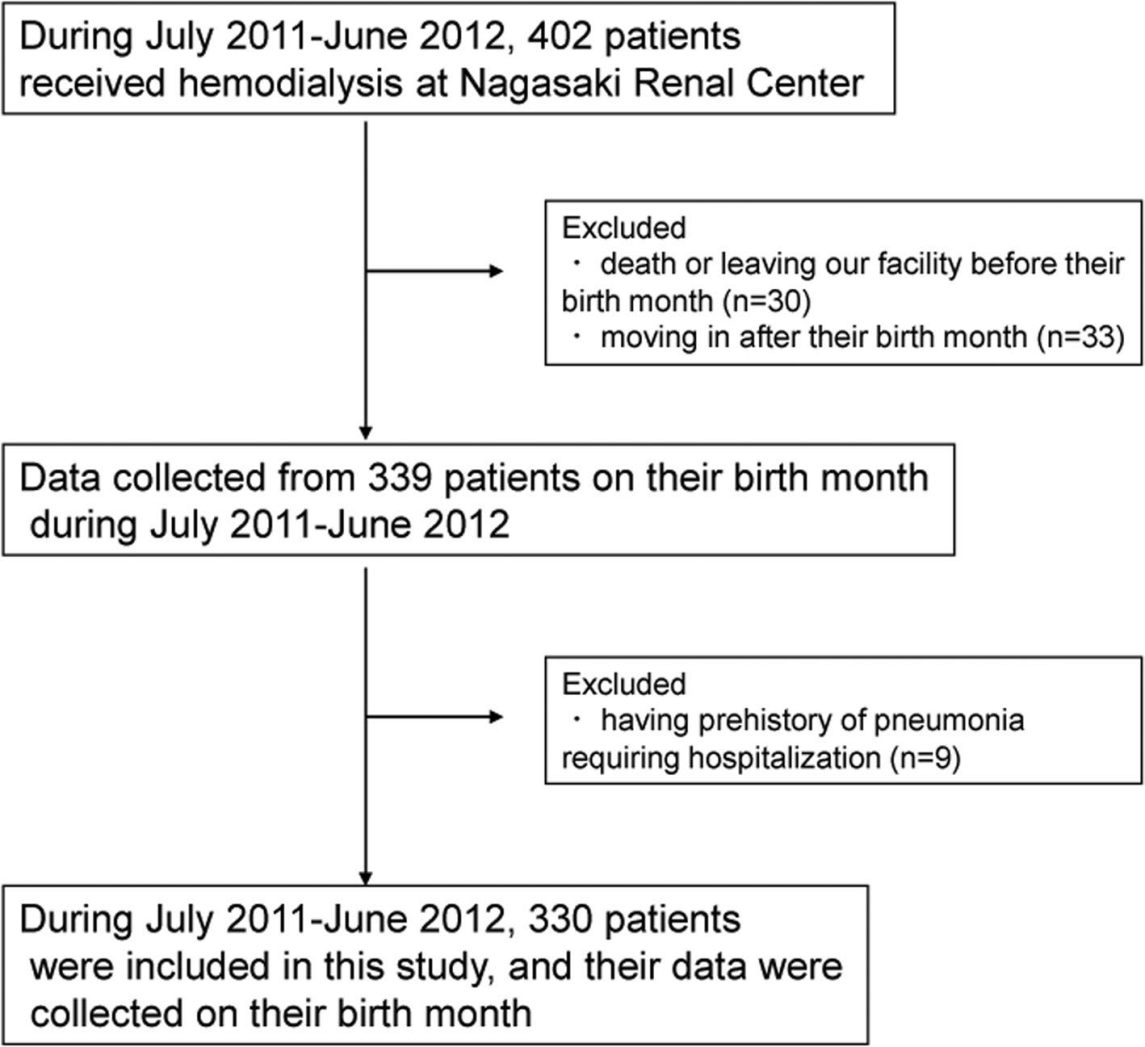
Patient flowchart

HD patients who were treated at the Nagasaki Renal Center between July 2011 and June 2012 were enrolled. To be included, patients of ≥ 20 years of age had to be on dialysis for more than three months. Patients who left the Nagasaki Renal Center or died before their birthdays during the inclusion period were excluded because the routine examinations were scheduled during their birth month. Patients with a history of pneumonia requiring hospitalization within one year before the inclusion period were also excluded. The patient selection flowchart is shown in Fig. 1. The observation period was between July 2011 and June 2021, and the follow-up continued to the date of incident pneumonia development requiring hospitalization, death, kidney transplantation, and leaving our hospital. The medical records and radiological findings were reviewed to confirm the diagnosis of pneumonia based on the following criteria: the presence of new infiltrates on chest X-ray or CT, symptoms of a lower respiratory tract infection, inflammatory findings in laboratory data, and antibiotic use. The diagnosis of pneumonia was made by attending physicians and confirmed by the investigators (Ko. Y., M. K., T. T., and Ka. Y.) who were in charge of recruitment for the study. The incidence of death was considered a competing event.

### Data collection

The data on patient characteristics, including age, sex, duration of dialysis, blood examinations, complications, and drugs, were obtained from medical records during their birth month. Missing data were excluded from the analysis. The incidence of pneumonia requiring hospitalization was determined based on medical records during the observation period while excluding cases of hospital-acquired pneumonia.

A ratio of 1:200 was used to convert darbepoetin alfa and epoetin beta pegol to epoetin alfa, as previously described [14]. The GNRI was calculated based on the patient’s serum albumin and body weight using the modified version proposed by Yamada et al. [11]: GNRI = (14.89 × albumin [g/dL]) + [41.7 × (body weight/ideal body weight); if body mass index (BMI) exceeded 22, body weight/ideal body = 1]. According to an age threshold of 65 years, the NRI was calculated as follows [12]: NRI = low BMI + low serum albumin level + abnormal serum total cholesterol level + low serum creatinine level (low BMI [≤ 20 kg/m^2^], yes = 3, no = 0; low serum albumin level [young < 3.7 g/dL; old < 3.5 g/dL], yes = 4, no = 0; abnormal serum total cholesterol level [low: < 130 mg/dL = 1, high: ≥ 220 mg/dL = 2], low = 1, high = 2, no = 0; low serum creatinine level [young female, < 9.7 mg/dL; old female, < 8.0 mg/dL; young male, < 11.6 mg/dL; old male, < 9.7 mg/dL], yes = 4, no = 0).

### Measurement of PMI

**Fig. 2:**
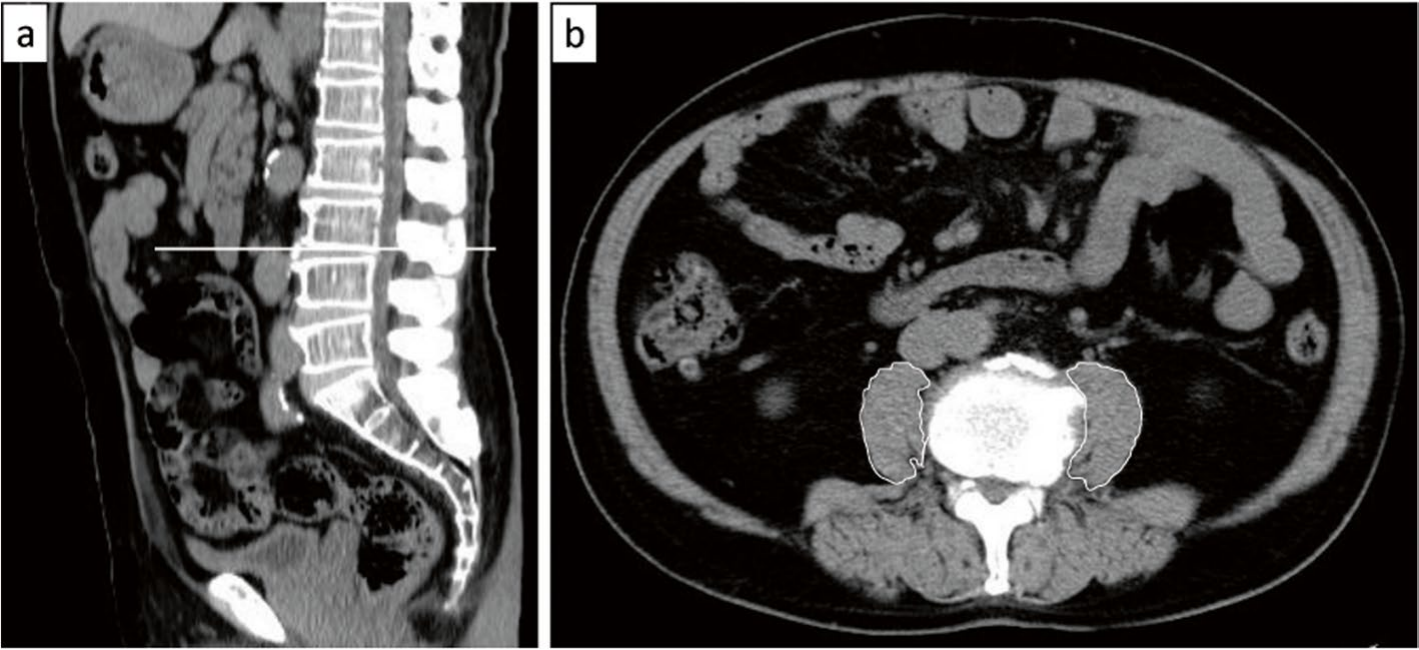
Measurement of psoas muscle area on the birth month’s CT scan. a. CT image showing the lower border of the third lumbar vertebra. b. The cross-sectional area of the bilateral psoas muscle was measured (manual trace method)

All CT scans (Bright Speed; GE Healthcare, Chicago, Illinois, IL, USA) were performed during the patients’ birth months between July 1, 2011 and June 30, 2012 and annually thereafter to detect acquired cystic kidney disease and renal cancer in HD patients. The cross-sectional area of the bilateral psoas muscle was measured at the lower border of the third lumbar vertebra (L3) using the lower trace method. In brief, psoas muscle mass was calculated by tracing the psoas muscles along the psoas marginal line visually using the Synapse (Fuji Film, Tokyo, Japan) radiological software [15–17]. The evaluation was performed by two experienced physicians (Ko. Y. and M. K.) who had been trained to use this approach by a radiologist. They had no information on the outcome of the participants in this study during tracing of the psoas muscles. If the psoas muscle marginal line was unclear due to compression fractures of the vertebrae, the final measurement was decided after discussion by the two physicians. Subsequently, the PMI was calculated as the cross-sectional area of the bilateral psoas muscle (lower border of L3)/height^2^ (mm^2^/m^2^) (Fig. 2). We divided the patients according to the quartile of the PMI (Q1–Q4) to evaluate tendency.

### Statistical analyses

Categorical variables are shown as numbers (%) and continuous variables as mean ± standard deviation. Non-normally distributed data are presented as median values with interquartile ranges. The t-test and Mann–Whitney U test were used to compare continuous variables between the two groups. The chi-squared test was used to compare the categorical variables. The analysis of variance and Kruskal–Wallis analysis were used to compare the ordinal variable among the four groups. Furthermore, the Kruskal–Wallis analysis of variance on ranks was used to compare the categorical variables. To determine the risk of contracting pneumonia according to the patients’ skeletal muscle mass, competing risk model and Cox proportional hazard model analyses were conducted. As the Cox proportional hazards analyses do not reflect the competing risk of death before the occurrence of an endpoint (and therefore, if a competing risk is present, the estimate of the remaining lifetime risk is biased), adjustment was made for this competing risk to yield an accurate estimate of the remaining lifetime risk of pneumonia. Model 1 calculated the risk adjusted for age, sex, dialysis duration, diabetes mellitus, history of stroke, and creatinine/standard deviation (SD). Model 2 calculated the risk adjusted for age, sex, duration of dialysis, diabetes mellitus, history of stroke, and PMI/SD. Model 3 calculated the risk adjusted for age, sex, duration of dialysis, diabetes mellitus, history of stroke, and GNRI/SD. Model 4 calculated the risk adjusted by age, sex, dialysis duration, diabetes mellitus, history of stroke, and NRI/SD. No missing parameters were used in multivariable analyses.

Simple correlation matrix was performed to determine the association between serum creatinine and PMI. Multiple logistic regression analyses were also performed. The constitutional parameters were age, sex, dialysis duration, and serum creatinine in Model 1; the residual parameters were added in Model 2 using the stepwise method (mixed method).” Adjusted odds ratios (ORs) and 95% confidence intervals (CIs) were calculated. Statistical significance was set at *p* < 0.05. Statistical analyses were mainly performed using JMP pro, version 15.0.0 software (SAS Institute Inc., Cary, North Carolina, NC, USA), and competing risk model analyses was performed using R software, version 4.1.1 (R project for statistical computing).

## Results

**Fig. 3:**
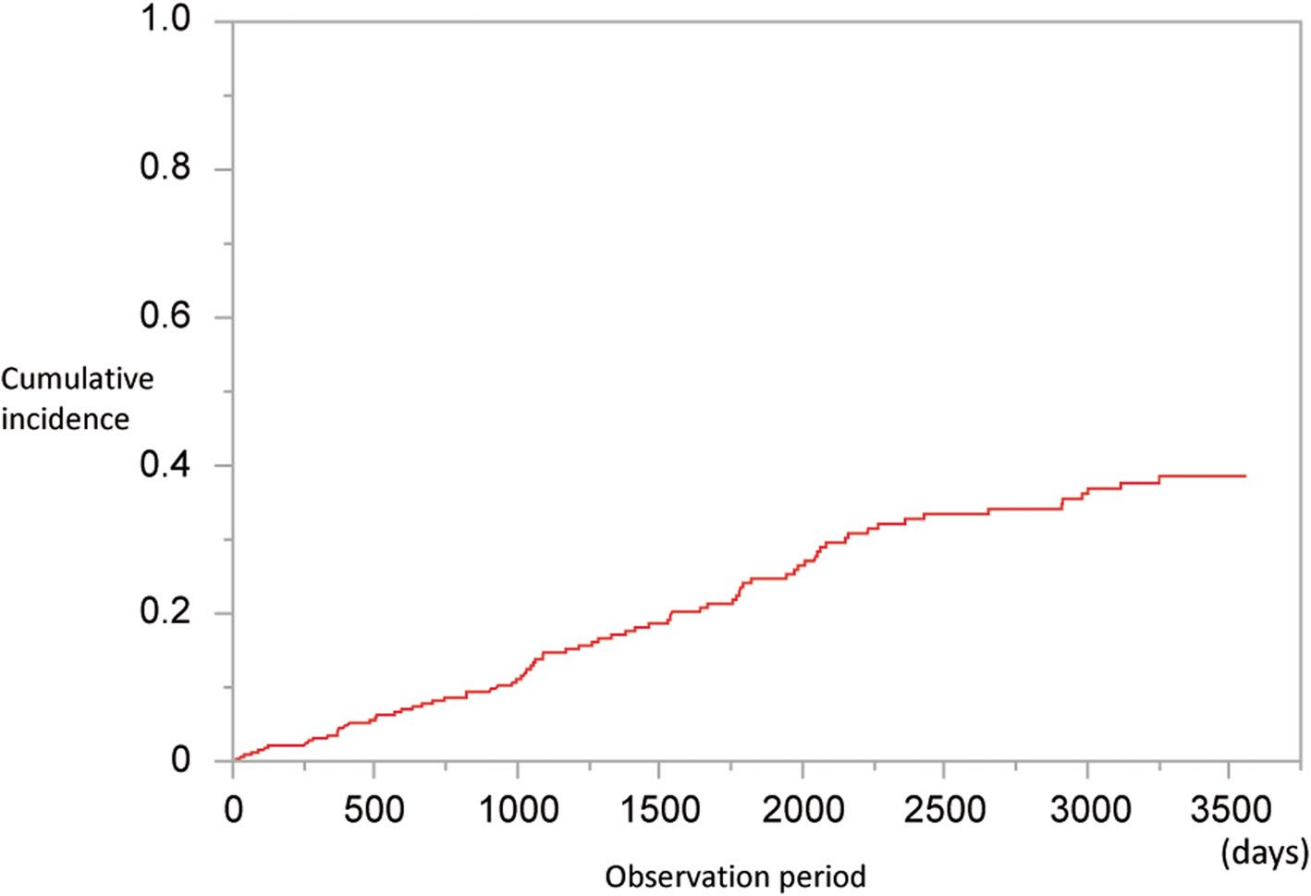
Cumulative incidence of pneumonia requiring hospitalization during the observation period

**Fig. 4:**
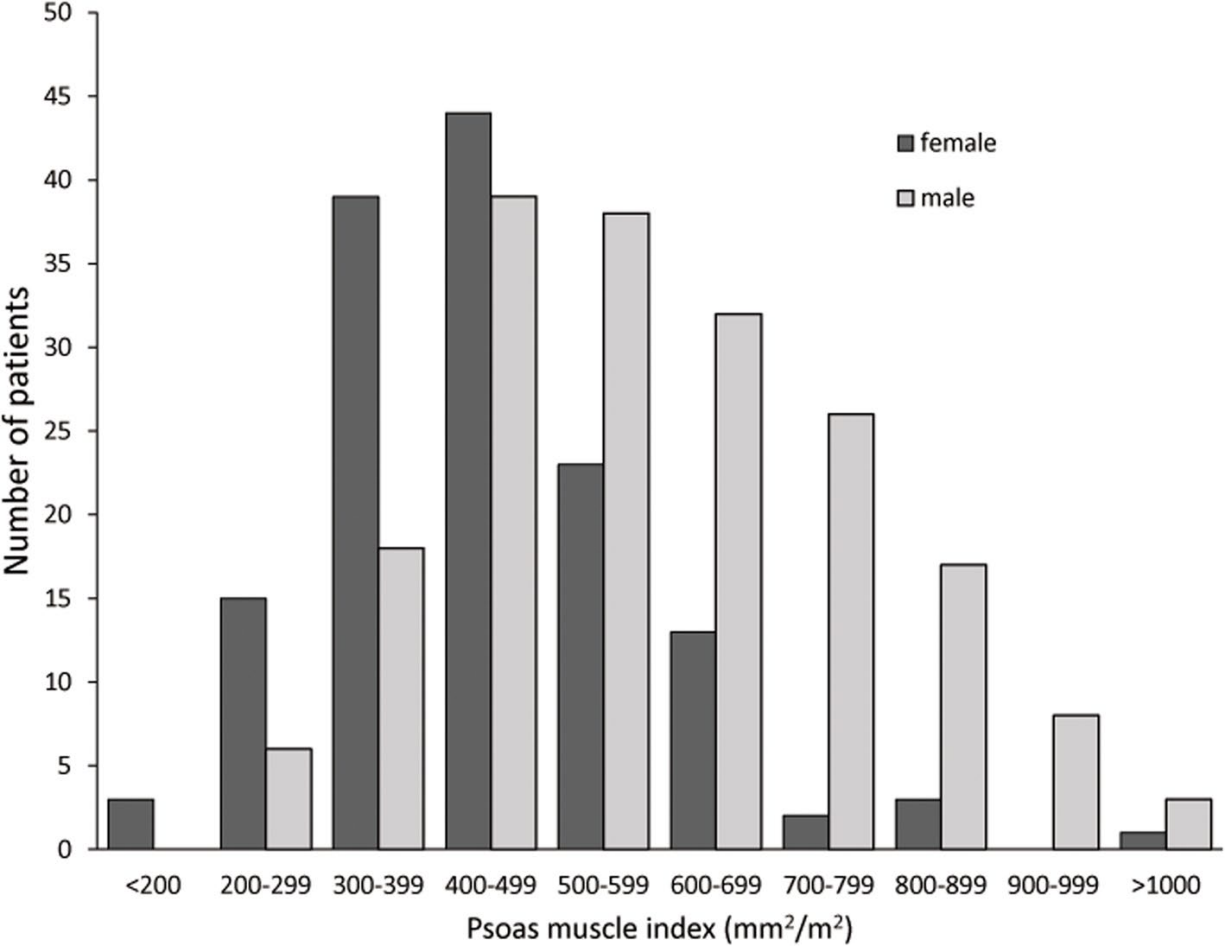
Distribution of the psoas muscle index according to sex

Between July 1, 2011, and June 30, 2012, 402 patients underwent HD at the Nagasaki Renal Center. Among them, 63 patients who either died or left our facility before their birth month (*n* = 30) or started care at our facility after their birth month (*n* = 33) were excluded. Moreover, nine patients were excluded because of a history of pneumonia within the preceding year. Therefore, 330 patients were included, of which 79 were hospitalized with pneumonia between July 2011 and June 2021. The cumulative incidence of pneumonia requiring hospitalization during the observation period is shown in Fig. 3. The average PMI was 600 ± 179 and 446 ± 141 mm^2^/m^2^ for men and women, respectively, exhibiting a significant difference (*p* < 0.001) (Fig. 4). Patient characteristics are shown in Table 1 and S1. Ferritin, transferrin saturation, and left ventricular ejection fraction had missing data; however, their proportions were within 5–10%. There were several differences between the four groups, including in age (p < 0.001) and serum creatinine levels (p < 0.001). During the observation period, 216 patients died, one patient underwent kidney transplantation, and two patients left our hospital (Table S2). Furthermore, the characteristics of pneumonia noted during the observation period are shown in Tables S3 and S4. All patients with pneumonia received antibiotics just before their death or for at least 5 days.

**Table 1:** Baseline patient characteristics

	Q1 PMI (160–403) (*n* = 83)	Q2 PMI (404–503) (*n* = 82)	Q3 PMI (503–633) (n = 83)	Q4 PMI (636–1097) (n = 82)	*P* value
Observation period^a^ (days)	1091 (425–3287)	1596 (862.5–3203)	1479 (723–3303)	2698 (1361–3446)	< 0.001
Age (years)	73.6 ± 11.2	69.4 ± 12.5	67.3 ± 12.4	58.7 ± 12.7	< 0.001
Female (%)	69.9	53.7	33.7	15.9	< 0.001
Duration of dialysis^a^ (months)	45 (14–108)	55 (22–118)	68 (23–124)	75 (31–147)	0.11
Dialysis time^a^ (h)	3 (3–4)	4 (3–4)	4 (3–4)	4 (3–4)	< 0.001
Kt/V	1.4 ± 0.5	1.4 ± 0.4	1.3 ± 0.3	1.3 ± 0.3	0.16
Ischemic heart disease (%)	33.7	39.0	33.7	26.8	0.43
Diabetes mellitus (%)	26.5	36.6	37.3	36.6	0.40
Cerebral hemorrhage (%)	3.6	9.8	3.6	8.5	0.23
Cerebral infarction (%)	25.3	24.4	26.5	17.1	0.48
Arteriosclerosis obliterans (%)	16.9	20.7	15.7	12.2	0.53
Cardiothoracic ratio (%)	54.8 ± 6.45	52.9 ± 4.9	50.7 ± 5.2	50 ± 4.8	< 0.001
Body weight (kg)	45.3 ± 7.6	49.9 ± 9.6	53.4 ± 9.7	60.2 ± 11.0	< 0.001
Body mass index (kg/m^2^)	18.9 ± 2.4	20.4 ± 2.8	21.1 ± 2.9	23.0 ± 3.7	< 0.001
Systolic blood pressure (mmHg)	147 ± 27	151 ± 24	147 ± 24	153 ± 23	0.17
Diastolic blood pressure (mmHg)	72 ± 11	77 ± 13	80 ± 14	83 ± 14	< 0.001
Left ventricular ejection fraction (%)	65 ± 11	65 ± 10	64 ± 9.7	66 ± 9.7	0.50
White blood cell (/μL)	5437 ± 2170	6047 ± 932	5264 ± 1912	4078 ± 1186	0.15
Hemoglobin (g/dL)	10.5 ± 1.5	10.8 ± 1.3	11.0 ± 1.3	11.0 ± 1.3	0.072
Ferritin^a^ (ng/mL)	62 (23–209)	59 (25.7–109)	45 (20–142)	57 (17–186)	0.89
TSAT (%)	23 ± 14	24 ± 11	24 ± 13	27 ± 14	0.42
Albumin (g/dL)	3.4 ± 0.4	3.6 ± 0.4	3.6 ± 0.4	3.8 ± 0.3	< 0.001
cCa (mg/dL)	9.2 ± 0.6	9.3 ± 0.8	9.2 ± 0.8	9.3 ± 0.7	0.38
P (mg/dL)	5.2 ± 1.3	5.4 ± 1.5	5.8 ± 1.5	6.1 ± 1.9	0.001
Intact-PTH^a^ (pg/mL)	61 (23–113)	54 (23–135)	80 (30–171)	112 (47–203)	0.004
ALP^a^ (IU/L)	255 (205–348)	260 (187–333)	266 (192–369)	221 (179–308)	0.057
BUN (mg/dL)	64 ± 20	68 ± 17	70 ± 17	71 ± 17	0.035
Creatinine (mg/dL)	8.3 ± 3.0	9.4 ± 2.9	11.0 ± 3.3	12.6 ± 3.0	< 0.001
Total cholesterol (mg/dL)	158 ± 36	166 ± 36	165 ± 3.6	157 ± 31	0.51
Triglycerides^a^ (mg/dL)	84 (65–121)	90 (63–117)	92 (70–133)	100 (65–147)	0.36
CRP^a^ (mg/dL)	0.23 (0.09–1.1)	0.16 (0.06–0.60)	0.14 (0.07–0.45)	0.18 (0.07–0.49)	0.13
Anti-platelet drugs (%)	39	41	41	34	0.76
Warfarin (%)	7.2	7.3	7.2	7.4	1.00
ESA^a^ (IU/week)	7500 (2000–10,000)	4500 (2000–9000)	4000 (2000–8000)	4000 (938–8000)	0.091
Iron (%)	25.3	14.6	20.5	17.1	0.34
Calcium carbonate (%)	32.5	47.6	56.6	54.9	< 0.001
Lanthanum carbonate (%)	19.3	35.4	27.7	45.1	0.003
Sevelamer (%)	1.2	2.5	3.6	4.9	0.55
Cinacalcet (%)	10.8	16.1	15.7	24.7	0.12
Vitamin D (%)	56.6	70.4	66.3	76.0	0.062
GNRI	85.8 ± 7.3	90.7 ± 7.3	92.3 ± 7.5	95.8 ± 6.4	< 0.001
NRI ^a^	8 (4–11)	5 (3–8)	4 (0–7)	3 (0–4)	< 0.001
Pneumonia (%)	26	37	19	13	0.001

One Way Analysis of variance and The Kruskal-Wallis Analysis of Variance were used to compare continuous variables. The Kruskal-Wallis Analysis of Variance on Ranks was used to compare the categorical variables. *TSAT* transferrin saturation, *cCa* corrected calcium, *P* phosphate, *ALP* alkaline phosphatase, *BUN* blood urea nitrogen, *CRP* C-reactive protein, *ESA* erythropoiesis-stimulating agents, *BMI* body mass index, *GNRI* geriatric nutritional risk index, *NRI* nutritional risk index for hemodialysis patients, *PMI* psoas muscle mass index. ^a^ median (interquartile range)

**Fig. 5:**
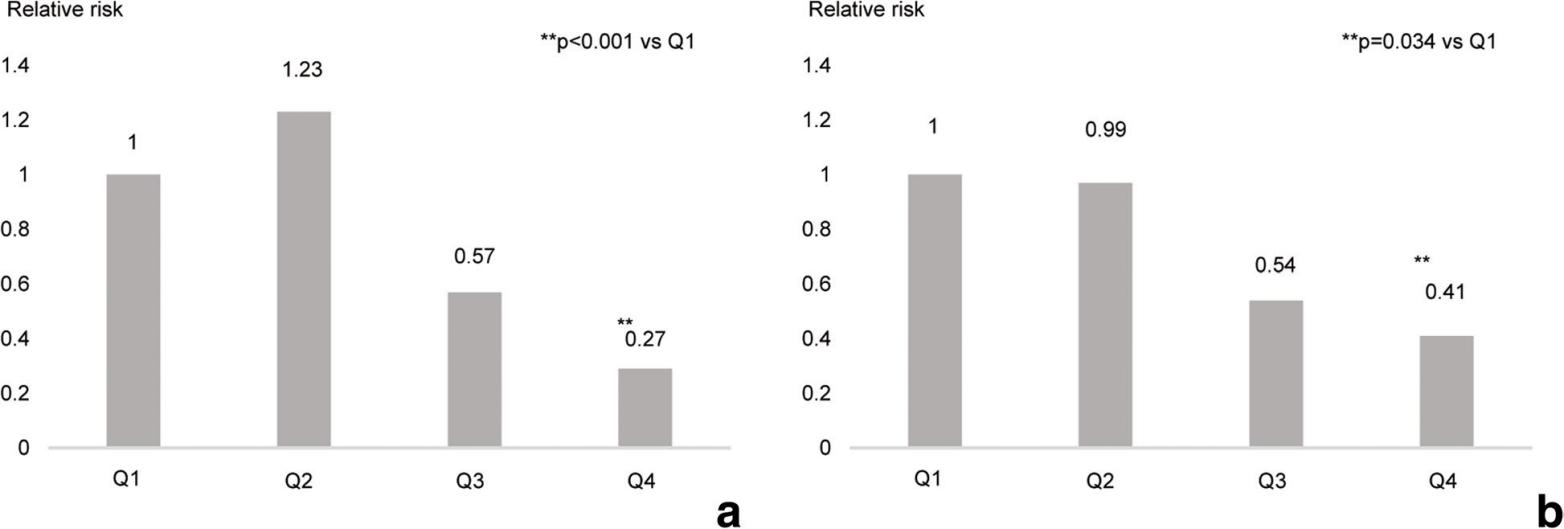
Associations between PMI and pneumonia development

Competing risk model analyses, revealed that a decreased PMI (*p* = 0.025) was a significant predictor of pneumonia development. Serum creatinine (*p* = 0.16), GNRI (*p* = 0.75), and NRI (*p* = 0.48) were not associated with pneumonia development (Table 2). In the Cox analysis, which did not consider the competitive risk, Cr, PMI, GNRI, and NRI were associated with pneumonia development (Table S5). Sensitivity analyses for sex and age were also conducted, and no difference was noted in the result (Table S6). Next, we analyzed the risk of mortality among Q1–Q4. In the unadjusted analysis, nonlinear associations were noted between the PMI and the outcome (Fig. 5a). In the adjusted analysis (for the exact same factors as those in the multivariable Cox regression analysis), a grade-dependent tendency was observed for the risk of developing pneumonia from Q1 to Q4 (Fig. 5b). Further, using the stepwise method, PMI was significantly correlated with the serum creatinine levels, and simple regression analysis was performed (β, 0.493; 95% confidence interval, 30.0–30.9; *p* < 0.001) (r^2^ = 0.244) (Table 3). Moreover, multiple regression analyses, which were performed using two models developed using the stepwise method, indicated that PMI was significantly correlated with the serum creatinine levels (β, 0.493; 95% confidence interval, 4.63–15.3; p < 0.001) (r^2^ = 0.476) (Table S7).

**Table 2:** Competing-Risk Cox regression analysis of the risk of pneumonia requiring hospitalization

	Model 1			Model 2			Model 3			Model 4		
	HR	95% CI	*P* value	HR	95% CI	*P* value	HR	95% CI	*P* value	HR	95% CI	*P* value
Age /year	1.03	1.01 to 1.06	0.017	1.03	1.00 to 1.05	0.027	1.04	1.02 to 1.07	0.017	1.04	1.01 to 1.07	0.004
Female vs male	0.68	0.38 to 1.16	0.15	0.66	0.38 to 1.12	0.12	0.77	0.46 to 1.31	0.34	0.76	0.45 to 1.30	0.32
Dialysis vintage /year	1.00	0.97 to 1.04	0.72	1.00	1.97 to 1.04	0.95	1.00	0.96 to 1.04	0.97	1.00	0.96 to 1.04	0.97
DM history	1.17	0.68 to 2.02	0.57	1.23	0.71 to 2.14	0.46	1.18	0.69 to 2.02	0.55	1.20	0.70 to 2.01	0.50
Stroke history	1.80	1.02 to 3.21	0.044	1.89	1.08 to 3.32	0.026	1.88	1.05 to 3.35	0.033	1.86	1.05 to 3.31	0.035
Creatinine /SD	0.78	0.56 to 1.10	0.16	–	–	–	–	–	–	–	–	–
PMI /SD	–	–	–	0.67	0.47 to 0.95	0.025	–	–	–	–	–	–
GNRI /SD	–	–	–	–	–	–	1.04	0.80 to 1.35	0.75	–	–	–
NRI /SD	–	–	–	–	–	–	–	–	–	1.10	0.84 to 1.45	0.48

The increase in the hazard ratio for one-unit change in the continuous variable. Model 1: adjusted for serum albumin and creatinine, Model 2: adjusted for the psoas muscle index, Model 3: adjusted for the geriatric nutritional risk index, and Model 4: adjusted for the psoas muscle mass index

*HR* hazard ratio, *95% CI* 95% confidence interval, *DM* diabetes mellitus, *SD* standard deviation, *PMI* psoas muscle mass index, *GNRI* geriatric nutritional risk index, *NRI* nutritional risk index for hemodialysis patients

**Table 3:** Relationship between the psoas muscle index and serum creatinine levels

	The psoas muscle index		
	β	95% CI	*P* value
Creatinine (mg/dL)	0.274	30.0 to 30.9	< 0.001
r^2^	0.244		

95% CI, 95% confidence interval

## Discussion

To our knowledge, this is the first study that showed the association between PMI and developing pneumonia in patients on HD. A low PMI was a significant predictor of pneumonia development, as indicated by Cox regression models that consider death as a competing risk. Conversely, low serum creatinine level, GNRI, and NRI were not associated with pneumonia development. Based on this study, since pneumonia is an important issue in patients undergoing HD, PMI would be able to contribute to clinical practice. From this point of view, the risk of pneumonia development can be assessed by the result of routine abdominal CT in HD patients.

Pneumonia in HD patients may be caused by immune deficiency [3], dysphagia [18], and sarcopenia [9]. Owing to the high susceptibility of HD patients to pneumonia, their pneumonia is included in the category of nursing and healthcare-associated pneumonia (NHCAP) by the Japanese guidelines [19]. Since pneumonia is also the most common infectious cause of death in HD patients [4], investigating its predictors would be useful for improving the prognosis in these patients.

In HD patients, uremia causes malnutrition and chronic inflammation, resulting in increased catabolism and decreased body stores of metabolic fuel (i.e., body protein and fat) [7, 20]; this condition is called protein-energy wasting [7, 20]. In addition, as the mean age of HD patients has increased due to global aging, loss of skeletal muscle mass and strength has become more common in HD patients [6]. Dual-energy X-ray absorptiometry and bioelectrical impedance analysis are the gold standards for accurately evaluating muscle mass [8, 21]; however, these methods are not readily available at HD facilities. CT-based evaluation of the psoas muscle cross-sectional area and subsequent calculation of PMI has shown good inter-rater reliability [16, 22]. Furthermore, PMI correlated with the skeletal muscle mass in dialysis patients [16, 23]. Therefore, annual CT scans performed to detect acquired cystic and malignant kidney disease are useful for HD patients [23, 24] and may be utilized for the assessment of trunk muscle mass.

Skeletal muscle mass has been shown to be associated with pneumonia in elderly individuals [25], and skeletal muscle strength and mass are correlated with swallowing muscle strength and dysphagia [26, 27]. Recently, the novel concept of sarcopenic dysphagia has been proposed [9]. It is highly likely that aspiration pneumonia is involved in several NHCAP, which include pneumonia in HD patients [19]. Although several factors are believed to contribute to pneumonia in HD patients, including aging and dysphagia due to post-stroke syndrome [2, 28], the factor most closely linked to aspiration pneumonia has not been elucidated [25]. We propose that the loss of skeletal muscle mass plays a major role in the occurrence of aspiration pneumonia. The correlation between low PMI and pneumonia noted in this study along with PMI’s known association with skeletal muscle mass [16, 23] corroborates the close association of skeletal muscle mass with pneumonia development in HD patients.

In this study, serum creatinine levels were not correlated with pneumonia development. Serum creatinine is a metabolic product of muscles, and serum creatinine levels in maintenance HD patients can reflect their skeletal muscle mass [29]. Additionally, serum creatinine levels are easy to measure in HD patients. Therefore, Cr was included in this study to confirm the relationship between skeletal muscle mass and pneumonia development in HD patients; however, the competing risk-adjusted Cox proportional hazard models showed that low serum creatinine levels were not a predictor of developing pneumonia. Although there was a weak correlation (r^2^ = 0.244) between PMI and serum creatinine levels, other factors, such as age and BMI, had to be added to make the correlation stronger (r^2^ = 0.476). This fact may also be affected that serum creatinine levels in HD patients were influenced not only by muscle mass but also by residual renal function, dietary intake, and dialysis efficiency.

Malnutrition plays an important role in skeletal muscle mass loss [6, 30]. Malnutrition in HD patients assessed by GNRI or NRI is associated with death [12, 31] as is skeletal muscle mass [6]. Moreover, malnutrition increases the risk of infectious disease in patients undergoing HD [32]. Therefore, we hypothesized that low nutrition is also associated with pneumonia development in HD patients; however, no significant association between GNRI or NRI and pneumonia development was found in the competing risk-adjusted Cox proportional hazard models. Since pneumonia in HD patients has been shown to have a causal association with cardiovascular disease and death [4], skeletal muscle mass indices, such as PMI, may be a more suitable prognostic marker in HD patients than nutritional indices, such as GNRI and NRI.

This study has several limitations. First, as this study was conducted in a non-randomized, retrospective manner at a single center, the results obtained may not be applicable to other populations. Second, although patients who had a history of pneumonia requiring hospitalization within the preceding year were excluded from this study, the complete, comprehensive history of pneumonia was not fully considered in this study; for example, patients who developed pneumonia that did not require hospitalization were included. Third, pathogenic bacteria in 73% of pneumonia cases were not confirmed, and the etiology of pneumonia, such as aspiration or atypical pneumonia, was not differentiated. This study may also include cases of pulmonary edema that were misdiagnosed as pneumonia and treated with antibiotics. Fourth, since October 2014, the administration of pneumococcal vaccines, such as pneumococcal polysaccharide vaccine 23, to elderly individuals (≥ 65 years) has been recommended in Japan, which might have affected the findings. Finally, data, including PMI and other nutritional indices, may change over time during the observation period; however, we used only a baseline value due to data availability.

## Conclusions

This study showed that unlike serum creatinine levels, GNRI, and NRI, a low PMI was a good predictor of pneumonia development in HD patients in the multivariable Cox proportional analysis that was adjusted for the competing risk. Serum creatinine levels were correlated with PMI although were not associated with pneumonia development in HD patients. Physicians should consider the skeletal muscle mass of HD patients while implementing the necessary measures to prevent the development of pneumonia. Further, these findings need to be verified in future rehabilitation and nutritional intervention studies. Moreover, the association between dysphagia and PMI in HD patients should be assessed in another study.

## Supplementary Information

**Additional file 1: Table S1**. Baseline patient characteristics.

**Additional file 2: Table S2**. Number of patients who were lost follow up, including death, kidney transplantation, and leaving hospital.

**Additional file 3: Table S3**. The Microorganisms isolated from HD patients with pneumonia.

**Additional file 4: Table S4**. Initial antibiotic treatment of HD patients with pneumonia.

**Additional file 5: Table S5**. Cox regression analysis of the risk of pneumonia requiring hospitalization.

**Additional file 6: Table S6**. Sensitivity Analyses for Factors associated with the psoas muscle index.

**Additional file 7: Table S7**. Factors associated with the psoas muscle index.

## Data Availability

The datasets used and/or analyzed during the current study are available from the corresponding author on reasonable request.

## Abbreviations

PMI: Psoas muscle index
HD: Hemodialysis
GNRI: Geriatric nutritional risk index
NRI: Nutritional risk index
BMI: Body mass index
SD: Standard deviation
IQR: Interquartile range
CI: Confidence interval
OR: Odds ratio
CT: Computed tomography
NHCAP: Nursing and healthcare-associated pneumonia
