# Supplementary material for: Association between the psoas muscle index and hospitalization for pneumonia in patients undergoing hemodialysis
Source: BMC Nephrol. 2021 Nov 27;22:394. doi: 10.1186/s12882-021-02612-7 (PMC8627609; doi:10.1186/s12882-021-02612-7)
Supplement: Supplementary file 1 — Additional file 1: Table S1. Baseline patient characteristics. [file 12882_2021_2612_MOESM1_ESM.docx]

**Table S1. Baseline patient characteristics**

|  | Pneumonia group  (n=79) | Non-Pneumonia group (n=251) | P value |
| --- | --- | --- | --- |
| Observation period^a^ (days) | 1170 (568–1972) | 1486 (609–3267) | 0.007 |
| Age (years) | 71.5±12.1 | 65.9±13.4 | 0.001 |
| Female (%) | 44.3 | 43.0 | 0.84 |
| Duration of dialysis^a^ (months) | 52 (22–61) | 61 (23–132) | 0.15 |
| Dialysis time^a^ (h) | 3.5 (3–4) | 4 (3–4) | 0.019 |
| Ischemic heart disease (%) | 39.2 | 31.5 | 0.21 |
| Diabetes mellitus (%) | 39.2 | 31.5 | 0.29 |
| Cerebral hemorrhage (%) | 8.9 | 5.6 | 0.31 |
| Cerebral infarction (%) | 29.1 | 21.5 | 0.17 |
| Arteriosclerosis obliterans (%) | 20.3 | 15.1 | 0.29 |
| Cardiothoracic ratio (%) | 52.7±5.4 | 52.0±5.8 | 0.24 |
| Body weight (kg) | 50.3±10.9 | 52.8±11.0 | 0.034 |
| Body mass index (kg/m^2^) | 20.8±3.5 | 20.9±3.3 | 0.81 |
| Systolic blood pressure (mmHg) | 150±25 | 149±24 | 0.95 |
| Diastolic blood pressure (mmHg) | 76±13 | 79±14 | 0.017 |
| Left ventricular ejection fraction (%) | 64±11 | 65±10 | 0.39 |
| White blood cell (/μL) | 5090$\pm$1720 | 5590$\pm$1730 | 0.030 |
| Hemoglobin (g/dL) | 10.8±1.4 | 10.8±1.3 | 0.71 |
| Ferritin^a^ (ng/mL) | 75 (25–202) | 50 (19–143) | 0.29 |
| TSAT (%) | 28±14 | 23±14 | 0.017 |
| Albumin (g/dL) | 3.5±0.4 | 3.6±0.4 | 0.28 |
| cCa (mg/dL) | 9.1±0.6 | 9.3±0.8 | 0.42 |
| P (mg/dL) | 5.3±1.3 | 5.7±1.7 | 0.19 |
| Intact-PTH^a^ (pg/mL) | 63 (21–116) | 77 (29–167) | 0.064 |
| ALP^a^ (IU/L) | 245 (192–333) | 245 (191–343) | 0.88 |
| BUN (mg/dL) | 66±18 | 69±18 | 0.21 |
| Creatinine (mg/dL) | 9.4±3.1 | 10.6±3.5 | 0.008 |
| Total cholesterol (mg/dL) | 159±36 | 163±38 | 0.44 |
| Triglycerides^a^ (mg/dL) | 81 (61–119) | 92 (65–134) | 0.22 |
| CRP^a^ (mg/dL) | 0.15 (0.05–0.41) | 0.18 (0.05–0.59) | 0.43 |
| Anti-platelet drugs (%) | 42 | 38 | 0.53 |
| Warfarin (%) | 10 | 6 | 0.28 |
| ESA^a^ (IU/week) | 4000 (2000–8000) | 4500 (2000–9000) | 0.21 |
| Iron (%) | 22.8 | 18.7 | 0.44 |
| Calcium carbonate (%) | 48 | 48 | 0.96 |
| Lanthanum carbonate (%) | 22 | 35 | 0.024 |
| Sevelamer (%) | 1.3 | 3.6 | 0.25 |
| Cinacalcet (%) | 10 | 19 | 0.058 |
| Vitamin D (%) | 70 | 65 | 0.59 |
| BMI | 20.8±3.5 | 20.8±3.3 | 0.81 |
| GNRI | 90.7±7.0 | 91.3±8.2 | 0.23 |
| NRI ^a^ | 4 (3-8) | 4 (1-8) | 0.21 |
| Cross-sectional area of the bilateral psoas muscle (mm^2^) | 1171±444 | 1405±533 | 0.001 |
| PMI (mm^2^/m^2^) | 481±163 | 550±183 | 0.003 |

The t-test and Mann-Whitney U test were used to compare continuous variables. The chi-square test was used to compare the categorical variables. TSAT, transferrin saturation; cCa, corrected calcium; P, phosphate; ALP, alkaline phosphatase; BUN, blood urea nitrogen; CRP, C-reactive protein; ESA, erythropoiesis-stimulating agents; BMI, body mass index; GNRI, geriatric nutritional risk index; NRI, nutritional risk index for hemodialysis patients; PMI, psoas muscle mass index

^a^ median (interquartile range)
