# Supplementary material for: Association between the psoas muscle index and hospitalization for pneumonia in patients undergoing hemodialysis
Source: BMC Nephrol. 2021 Nov 27;22:394. doi: 10.1186/s12882-021-02612-7 (PMC8627609; doi:10.1186/s12882-021-02612-7)
Supplement: Supplementary file 2 — Additional file 2: Table S2. Number of patients who were lost follow up, including death, kidney transplantation, and leaving hospital. [file 12882_2021_2612_MOESM2_ESM.docx]

**Table S2. Number of patients who were lost follow up, including death, kidney transplantation, and leaving hospital.**

|  | Pneumonia group (n=79) | Non-pneumonia group (n=251) |
| --- | --- | --- |
| Death | 63 (80%) | 153 (61%) |
| Kidney transplantation | 0 (0%) | 1 (0.4%) |
| Leaving hospital | 0 (0%) | 2 (0.8%) |
