# Supplementary material for: Association between the psoas muscle index and hospitalization for pneumonia in patients undergoing hemodialysis
Source: BMC Nephrol. 2021 Nov 27;22:394. doi: 10.1186/s12882-021-02612-7 (PMC8627609; doi:10.1186/s12882-021-02612-7)
Supplement: Supplementary file 3 — Additional file 3: Table S3. The Microorganisms isolated from HD patients with pneumonia. [file 12882_2021_2612_MOESM3_ESM.docx]

**Table S3. The Microorganisms isolated from HD patients with pneumonia**

| Microorganisms | Pneumonia (n=79) |
| --- | --- |
| Klebsiella pneumoniae | 5 (6.3％) |
| MRSA | 4 (5.1％) |
| Pseudomonas aeruginosa | 3 (3.8％) |
| Escherichia coli | 3 (3.8％) |
| MSSA | 1 (1.3％) |
| MRSE | 1 (1.3％) |
| Enterobacter aerogenes | 1 (1.3％) |
| Moraxella catarrhalis | 1 (1.3％) |
| Stenotrophomonas maltophilia | 1 (1.3％) |
| Citrobacter freundii | 1 (1.3％) |
| Enterococcus faecalis | 1 (1.3％) |
| Oral bacteria | 17 (21.5％) |
| No organism identified | 8 (10.1％) |
| No specimen submitted | 33 (41.8％) |

MRSA; methicillin-resistant Staphylococcus aureus; MSSA, methicillin-sensitive Staphylococcus aureus; MRSE, Methicillin-resistant Staphylococcus epidermidis.
