# Supplementary material for: Association between the psoas muscle index and hospitalization for pneumonia in patients undergoing hemodialysis
Source: BMC Nephrol. 2021 Nov 27;22:394. doi: 10.1186/s12882-021-02612-7 (PMC8627609; doi:10.1186/s12882-021-02612-7)
Supplement: Supplementary file 4 — Additional file 4: Table S4. Initial antibiotic treatment of HD patients with pneumonia. [file 12882_2021_2612_MOESM4_ESM.docx]

**Table S4. Initial antibiotic treatment of HD patients with pneumonia**

| Treatment regimens | Pneumonia (n=79) |
| --- | --- |
| Tazobactam/Piperacillin | 27 (34.2％) |
| Sulbactam/Ampicillin | 23 (29.1％) |
| Doripenem | 22 (27.8％) |
| Ceftriaxone | 4 (5.1％) |
| Doripenem + Clindamycin | 1 (1.3％) |
| Doripenem + Ciprofloxacin | 1 (1.3％) |
| Tazobactam/Piperacillin + Ciprofloxacin | 1 (1.3％) |
