# Supplementary material for: Association between the psoas muscle index and hospitalization for pneumonia in patients undergoing hemodialysis
Source: BMC Nephrol. 2021 Nov 27;22:394. doi: 10.1186/s12882-021-02612-7 (PMC8627609; doi:10.1186/s12882-021-02612-7)
Supplement: Supplementary file 5 — Additional file 5: Table S5. Cox regression analysis of the risk of pneumonia requiring hospitalization. [file 12882_2021_2612_MOESM5_ESM.docx]

**Table S5. Cox regression analysis of the risk of pneumonia requiring hospitalization**

|  | Model 1 | | | Model 2 | | | Model 3 | | | Model 4 | | |
| --- | --- | --- | --- | --- | --- | --- | --- | --- | --- | --- | --- | --- |
|  | HR | 95% CI | P value | HR | 95% CI | P value | HR | 95% CI | P value | HR | 95% CI | P value |
| Age /year | 1.06 | 1.04 to 1.09 | <0.001 | 1.07 | 1.04 to 1.10 | <0.001 | 1.07 | 1.05 to 1.10 | <0.001 | 1.07 | 1.05 to 1.10 | <0.001 |
| Female vs male | 0.59 | 0.37 to 0.96 | 0.034 | 0.63 | 0.39 to 1.02 | 0.06 | 0.83 | 0.52 to 1.31 | 0.43 | 0.85 | 0.54 to 1.33 | 0.47 |
| Dialysis vintage /year | 1.00 | 1.00 to 1.00 | 0.30 | 1.00 | 1.00 to 1.00 | 1.00 | 1.00 | 1.00 to 1.00 | 0.96 | 1.00 | 1.00 to 1.00 | 0.64 |
| DM history | 1.38 | 0.86 to 2.23 | 0.19 | 1.55 | 0.95 to 2.50 | 0.080 | 1.44 | 0.89 to 2.33 | 0.13 | 1.49 | 0.92 to 2.41 | 0.10 |
| Stroke history | 1.82 | 1.08 to 3.06 | 0.030 | 1.94 | 1.16 to 3.24 | 0.016 | 1.92 | 1.15 to 3.21 | 0.018 | 1.75 | 1.03 to 2.95 | 0.045 |
| Creatinine(mg/dl)/SD | 0.52 | 0.39 to 0.73 | <0.001 | - | - | - | - | - | - | - | - | - |
| PMI (mm^2^/m^2)^/SD | - | - | - | 0.62 | 0.45 to 0.84 | 0.002 | - | - | - | - | - | - |
| GNRI/SD | - | - | - | - | - | - | 0.72 | 0.55 to 0.96 | 0.024 | - | - | - |
| NRI/SD | - | - | - | - | - | - | - | - | - | 1.46 | 1.14 to 1.87 | 0.003 |

The increase in the hazard ratio for one-unit change in the continuous variable. Model 1: adjusted for serum albumin and creatinine, Model 2: adjusted for the psoas muscle index, Model 3: adjusted for the geriatric nutritional risk index, and Model 4: adjusted for the psoas muscle mass index

HR, hazard ratio; 95% CI, 95% confidence interval; DM, diabetes mellitus; SD, standard deviation; PMI, psoas muscle mass index; GNRI, geriatric nutritional risk index; NRI, nutritional risk index for hemodialysis patients.
