# Supplementary material for: Association between the psoas muscle index and hospitalization for pneumonia in patients undergoing hemodialysis
Source: BMC Nephrol. 2021 Nov 27;22:394. doi: 10.1186/s12882-021-02612-7 (PMC8627609; doi:10.1186/s12882-021-02612-7)
Supplement: Supplementary file 6 — Additional file 6: Table S6. Sensitivity Analyses for Factors associated with the psoas muscle index. [file 12882_2021_2612_MOESM6_ESM.docx]

**Table S6 Sensitivity Analyses for Factors associated with the psoas muscle index**

|  | All (330) | | | Man (187) | | | Woman (143) | | |
| --- | --- | --- | --- | --- | --- | --- | --- | --- | --- |
|  | HR | 95% CI | P value | HR | 95% CI | P value | HR | 95% CI | P value |
| Age /year | 1.07 | 1.04 to 1.10 | <0.001 | 1.08 | 1.04 to 1.11 | <0.001 | 1.06 | 1.02 to 1.10 | 0.001 |
| Dialysis vintage /year | 1.00 | 0.97 to 1.03 | 0.95 | 1.01 | 0.96 to 1.06 | 0.63 | 0.98 | 0.93 to 1.02 | 0.50 |
| DM history | 1.57 | 0.97 to 2.54 | 0.069 | 1.51 | 0.80 to 2.84 | 1.54 | 1.44 | 0.72 to 3.32 | 0.28 |
| Stroke history | 2.06 | 1.24 to 3.43 | 0.005 | 1.65 | 0.83 to 3.26 | 0.17 | 2.68 | 1.23 to 5.87 | 0.022 |
| PMI /SD | 0.69 | 0.52 to 0.93 | 0.013 | 0.67 | 0.03 to 0.86 | 0.033 | 0.50 | 0.26 to 0.95 | 0.023 |

|  | All (330) | | | <65 y/o (147) | | | ≧65 y/o (183) | | |
| --- | --- | --- | --- | --- | --- | --- | --- | --- | --- |
|  | HR | 95% CI | P value | HR | 95% CI | P value | HR | 95% CI | P value |
| Female vs male | 0.63 | 0.39 to 1.02 | 0.058 | 0.71 | 0.29 to 1.74 | 0.45 | 0.51 | 0.28 to 0.93 | 0.029 |
| Dialysis vintage /year | 0.98 | 0.94 to 1.01 | 0.11 | 1.02 | 0.97 to 1.07 | 0.40 | 0.96 | 0.91 to 1.01 | 0.12 |
| DM history | 1.46 | 0.90 to 2.37 | 0.12 | 2.13 | 0.81 to 5.56 | 1.13 | 1.23 | 0.69 to 2.19 | 0.48 |
| Stroke history | 1.98 | 1.19 to 3.31 | 0.013 | 3.52 | 1.44 to 8.59 | 0.010 | 1.30 | 0.67 to 2.49 | 0.45 |
| PMI /SD | 0.49 | 0.36 to 0.64 | <0.001 | 0.64 | 0.41 to 0.99 | 0.046 | 0.46 | 0.30 to 0.69 | <0.001 |

The increase in the hazard ratio for one-SD change in the continuous variable. Model 1: adjusted for serum albumin and creatinine, Model 2: adjusted for the psoas muscle index, Model 3: adjusted for the geriatric nutritional risk index, and Model 4: adjusted for the psoas muscle mass index

HR, hazard ratio; 95% CI, 95% confidence interval; DM, diabetes mellitus; SD, standard deviation; PMI, psoas muscle mass index; GNRI, geriatric nutritional risk index; NRI, nutritional risk index for hemodialysis patients.
