# Supplementary material for: Association between the psoas muscle index and hospitalization for pneumonia in patients undergoing hemodialysis
Source: BMC Nephrol. 2021 Nov 27;22:394. doi: 10.1186/s12882-021-02612-7 (PMC8627609; doi:10.1186/s12882-021-02612-7)
Supplement: Supplementary file 7 — Additional file 7: Table S7. Factors associated with the psoas muscle index. [file 12882_2021_2612_MOESM7_ESM.docx]

**Table S7. Factors associated with the psoas muscle index**

|  | Model 1 | | | Model 2 | | |
| --- | --- | --- | --- | --- | --- | --- |
|  | β | 95% CI | P  Value | β | 95% CI | P  value |
| Age (years) | -0.265 | -4.99 to -2.20 | <0.001 | -0.183 | -3.97 to -1.37 | <0.001 |
| Female (yes/no) | -0.288 | -69.3 to -35.6 | <0.001 | -0.295 | -68.3 to -37.0 | <0.001 |
| Dialysis vintage (years) | -0.061 | -3.42 to 0.68 | 0.19 | 0.005 | -1.85 to 1.95 | 0.96 |
| Creatinine (mg/dL) | 0.274 | 8.7 to 20.1 | <0.001 | 0.195 | 4.6 to 15.3 | <0.001 |
| BMI (kg/m^2^) |  |  |  | 0.338 | 13.5 to 22.7 | <0.001 |
| CRP (mg/dL) |  |  |  | -0.117 | -23.7 to -4.0 | 0.005 |
| r^2^ | 0.368 | | | 0.476 | | |

Model 1: included clinically important factors for muscle mass; Model 2: Model 1 + parameters added included using the stepwise method (Akaike method).

95% CI, 95% confidence interval; BMI, body mass index; CRP, C-reactive protein
